# Primary diffuse large B-cell lymphoma of the rectus abdominis muscle: a presumed primary case report and literature review

**DOI:** 10.3389/fonc.2026.1837952

**Published:** 2026-06-02

**Authors:** Xuejuan Duan, Xiangyong Yue, Jianlei Cao, Yadong Liu, Jun Zhang, Yuguang Shang

**Affiliations:** 1 Department of Radiation Oncology, Fourth Hospital of Hebei Medical University, Shijiazhuang, China; 2 Department of Oncology, Hebei General Hospital, Shijiazhuang, China; 3 Department of Stomatology, Hebei General Hospital, Shijiazhuang, China

**Keywords:** diffuse large B-cell lymphoma, extranodal lymphoma, lymphoma, rectus abdominis muscle, R-THP-COP regimen

## Abstract

Primary diffuse large B-cell lymphoma (DLBCL) of the skeletal muscle is an extremely rare subtype of extranodal non-Hodgkin lymphoma, and primary DLBCL of the rectus abdominis muscle is even rarer with few reports in the literature. Due to the lack of specific clinical manifestations and imaging features, it is easily misdiagnosed, leading to delayed treatment. We herein report an 85-year-old female patient who presented with a painful mass in the right lower abdominal wall. Preoperative contrast-enhanced computed tomography (CT) showed an irregular soft-tissue mass in the right abdominal wall with unclear boundaries abutting adjacent small-bowel loops. Tumour markers were within normal limits; complete blood count, lactate dehydrogenase, β2-microglobulin and peripheral-blood flow cytometry were unremarkable, and contrast-enhanced CT of the chest and brain magnetic resonance imaging (MRI) revealed no other lesions. Tumour markers were within normal limits. Contrast-enhanced ultrasound suggested a hypervascular malignant lesion. The diagnosis of a presumed primary DLBCL of the rectus abdominis muscle (non-germinal centre B-cell-like subtype) was confirmed by ultrasound-guided needle biopsy and immunohistochemistry. The patient received 4 cycles of an attenuated R-THP-COP regimen (rituximab, pirarubicin [tetrahydropyranyl adriamycin, THP, substituted for doxorubicin to reduce cardiotoxicity], cyclophosphamide, vincristine and prednisone). Partial response was achieved after 2 cycles, and complete radiological response on contrast-enhanced CT was obtained after 4 cycles. The patient remained free of reported abdominal symptoms during telephone follow-up (no further imaging surveillance was performed) and died of cerebral haemorrhage 3 years later, from a cause unrelated to the lymphoma. Primary muscular DLBCL is highly aggressive and easily misdiagnosed. Pathological biopsy combined with immunohistochemistry is the gold standard for diagnosis. In this single elderly patient, an attenuated R-THP-COP regimen was well tolerated and produced a sustained complete clinical response, suggesting that an individualised, biopsy-guided, chemotherapy-based approach may be a reasonable option for similarly localised disease in elderly patients; this observation, however, requires confirmation in larger series. This case enriches the clinical data of this rare disease and provides a reference for clinical diagnosis and treatment.

## Introduction

Diffuse large B-cell lymphoma (DLBCL) is the most common aggressive subtype of non-Hodgkin lymphoma (NHL), characterized by remarkable clinical and biological heterogeneity (1, 2). Although DLBCL typically originates in the lymph nodes, approximately 25% to 40% of cases present as primary extranodal disease, with the exact proportion varying across populations and study designs (e.g., 27% reported in a recent three-year tertiary-centre series (3)). Common extranodal sites include the gastrointestinal tract, central nervous system, bone, and head and neck regions (4). However, primary lymphoma of the skeletal muscle system is extremely rare, accounting for less than 1% of all extranodal lymphomas and fewer than 0.5% of all malignant soft tissue tumours (5). Among these rare cases of muscular involvement, primary DLBCL of the rectus abdominis muscle is particularly uncommon, and to the best of our knowledge has not been reported previously.

The diagnosis of primary rectus abdominis lymphoma is greatly challenging during initial clinical evaluation due to the wide spectrum of lesions in the abdominal wall soft tissues and the lack of specific clinical manifestations and imaging features. Patients usually present with an insidious, localized, painless or mildly tender mass (6). On imaging examinations such as computed tomography (CT) or magnetic resonance imaging (MRI), the findings are frequently indistinguishable from more common disorders, including benign tumours, soft tissue sarcomas, abdominal wall abscesses, hematomas, or inflammatory myopathies. This non-specificity often leads to clinical misdiagnosis or unnecessary extensive surgical resection, thereby delaying the optimal systemic treatment dominated by systemic chemotherapy for lymphoma (7). Therefore, early identification of such rare and highly aggressive lymphomas and accurate histopathological diagnosis are crucial in elderly patients with soft tissue masses.

Given the rarity and high complexity in the differential diagnosis of primary rectus abdominis DLBCL, we herein report a rare case of an 85-year-old female presenting with a mass in the right lower abdominal wall. The patient was admitted due to an abdominal wall mass with aggravated pain. The final diagnosis of a presumed primary non-Hodgkin diffuse large B-cell lymphoma (non-germinal centre B-cell-like/activated B-cell subtype) of the rectus abdominis muscle was confirmed by ultrasound-guided core needle biopsy combined with detailed immunohistochemical analysis. This study aims to comprehensively analyse the clinical manifestations, imaging features, and detailed immunophenotype of this case, further enhance clinicians’ awareness of this rare extranodal lymphoma at this site, and provide evidence-based references for optimizing the standardized diagnostic procedure of similar atypical soft tissue tumours.

## Case presentation

**Figure 1:**
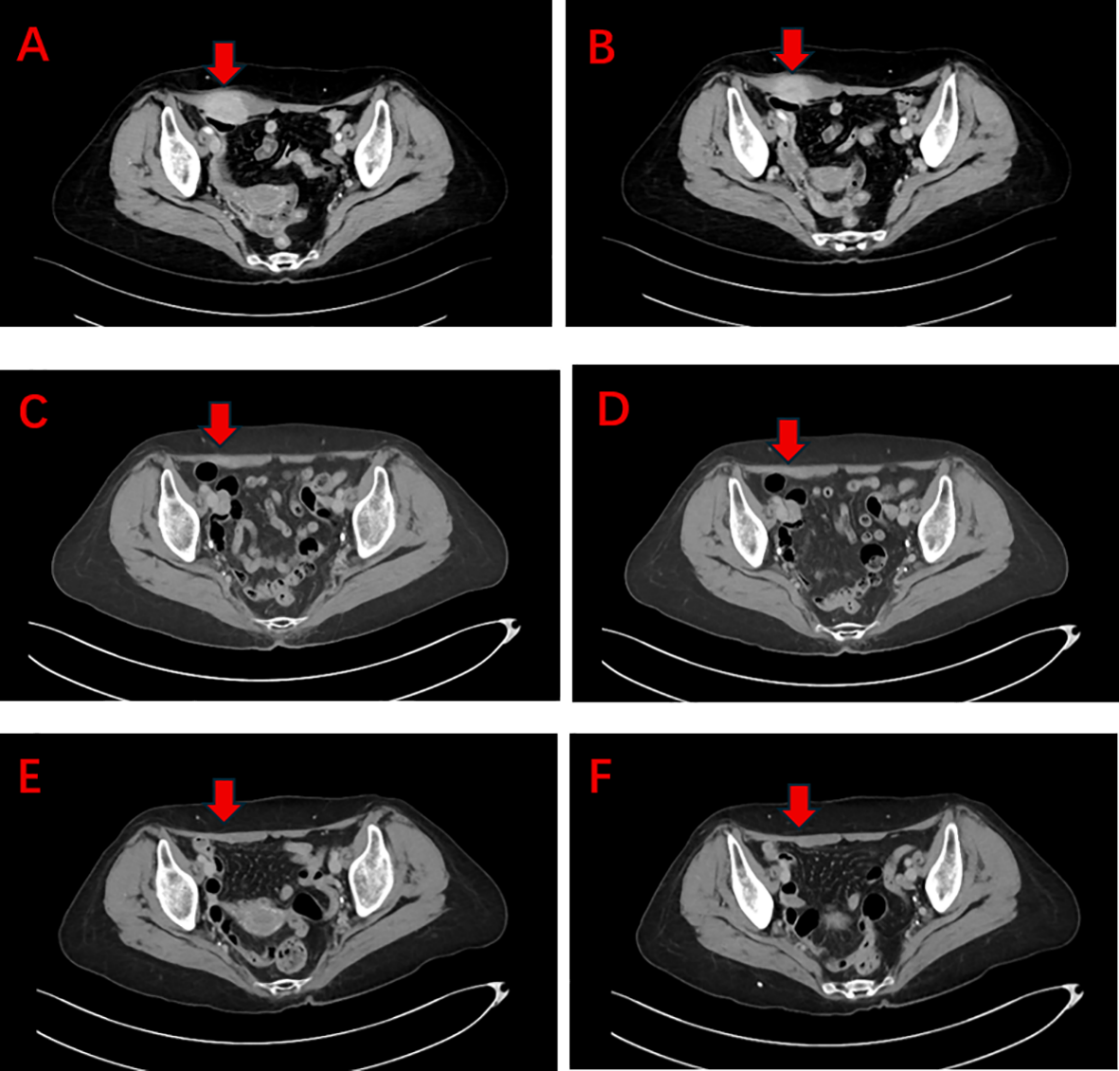
**(A, B)** CT findings at initial diagnosis. **(C, D)** Follow-up after 2 cycles of treatment, with therapeutic efficacy evaluated as partial response (PR). **(E, F)** Follow-up after 4 cycles of treatment, with therapeutic efficacy evaluated as complete response (CR).

An 85-year-old female patient accidentally found a mass in the right lower abdominal wall on June 15, 2021. She experienced aggravated pain and presented to our hospital on June 30, 2021. Physical examination revealed a palpable mass in the right lower abdominal wall, approximately 4 cm in size, with poor mobility, mild tenderness, no redness or swelling, and normal skin temperature. She was initially admitted to the general-surgery service, where the working clinical impression was a primary abdominal-wall soft-tissue tumour (e.g., desmoid tumour, sarcoma) or a complicated hernia, and surgical exploration with intraoperative frozen section was the initial plan, in accordance with local practice for palpable solid abdominal-wall masses. Preoperative pelvic contrast-enhanced computed tomography (CT) showed an iso-attenuating mass in the soft tissues of the right pelvic wall with marked heterogeneous enhancement and ill-defined borders, measuring approximately 41 mm × 31 mm × 42 mm. The lesion was poorly demarcated from some small bowel loops in the pelvic cavity, with thickening of the small intestinal wall (**Figures 1A, B**). Given the infiltrative borders and the close relationship of the mass to small-bowel loops — which substantially raised the risk of inadvertent enterotomy and incomplete oncological clearance — a multidisciplinary discussion (general surgery, radiology, pathology, oncology) recommended deferring surgery in this 85-year-old patient and obtaining tissue diagnosis by image-guided core-needle biopsy first.

**Figure 2:**
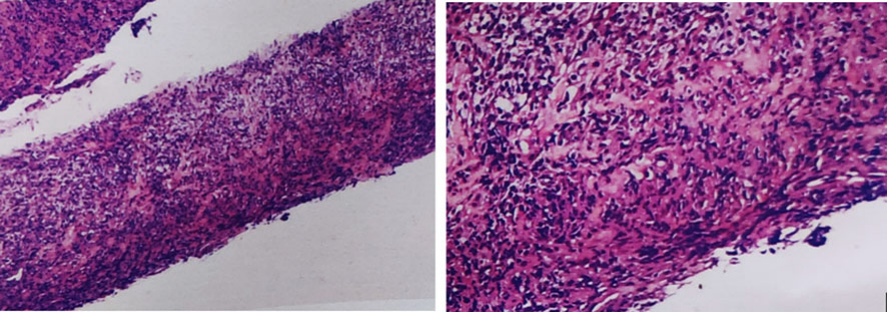
Pathological findings of ultrasound-guided needle biopsy of the abdominal wall muscle, consistent with diffuse large B-cell lymphoma (DLBCL).

Further examinations showed that tumour markers, including carbohydrate antigen 125 (CA125), carbohydrate antigen 153 (CA153), carbohydrate antigen 199 (CA199), neuron-specific enolase (NSE), cytokeratin 19 fragment (CYFRA21-1), alpha-fetoprotein (AFP), human chorionic gonadotropin (HCG), and squamous cell carcinoma antigen (SCC), were all within normal ranges. Complete blood count, peripheral-blood smear, lactate dehydrogenase (LDH), β2-microglobulin and peripheral-blood flow cytometry were also unremarkable. Contrast-enhanced CT of the chest and abdomen and contrast-enhanced MRI of the brain were obtained as part of the staging work-up and revealed no other lesions or pathological lymphadenopathy. 18F-FDG PET/CT and bone marrow biopsy were offered but declined by the patient and her family on financial and age-related grounds. Contrast-enhanced ultrasound revealed a hypervascular lesion in the muscular layer of the right lower abdominal wall, highly suggestive of malignancy. Ultrasound-guided needle biopsy was performed. Histopathology was consistent with non-Hodgkin diffuse large B-cell lymphoma, not otherwise specified (NOS), of non-germinal centre/activated B-cell origin (**Figure 2**). Immunohistochemical staining: CKpan (−), Vimentin (+), LCA (+), CD3 (−), CD20 (+), CD43 (−), CD79α (+), PAX-5 (+), CD21 (−), CD30 (−), P53 (approximately 70% +), CD10 (−), MUM1 (+), CD5 (−), Cyclin D1 (−), ALK (−), EMA (−), c-MYC (approximately 40% +), Bcl-6 (+), Bcl-2 (+), CD23 (−), Ki-67 (approximately 90% +). (**Supplementary Materials**) EBER *in situ* hybridization was negative. On the basis of a single anatomically isolated muscular lesion, negative comprehensive conventional staging, and unremarkable haematological parameters, the patient was clinically classified as cIE-stage presumed primary rectus-abdominis DLBCL.

**Figure 3:**
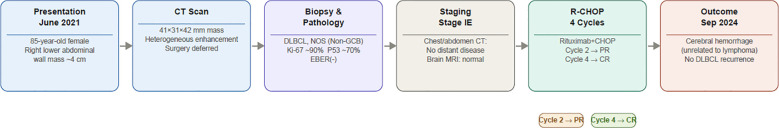
Flowchart of the diagnosis and treatment process for the patient.

Given the advanced age, localized lesion, and absence of distant metastasis on conventional imaging, the patient received 4 cycles of an attenuated R-THP-COP regimen — rituximab 0.5 g on day 0; cyclophosphamide 1000 mg on day 1; vincristine 1 mg on day 1; pirarubicin (tetrahydropyranyl adriamycin, THP) 30 mg on day 1; and prednisone — every 21 days. Pirarubicin (THP) was substituted for doxorubicin to reduce the risk of anthracycline-related cardiotoxicity in this 85-year-old patient; previous prospective and retrospective studies have shown that R-THP-COP achieves response and survival outcomes comparable to R-CHOP in elderly DLBCL patients with a more favourable cardiac safety profile. After 2 cycles, the efficacy evaluation by contrast-enhanced abdominal CT was partial response (PR) (**Figures 1C, D**). After 4 cycles, the evaluation by contrast-enhanced abdominal CT was complete response (CR), with complete resolution of the right rectus-abdominis mass (**Figures 1E, F**). In the absence of 18F-FDG PET/CT, this represents an anatomical (rather than metabolic) complete response. After CR, the patient declined further imaging surveillance for personal and economic reasons. She was followed by telephone every 3–6 months for a total of three years; these contacts confirmed only the absence of reported abdominal pain or new symptoms and cannot exclude asymptomatic recurrence. The patient did not undergo regular follow-up thereafter. Intermittent telephone follow-up showed stable disease without abdominal pain or other discomforts. The patient died of cerebral haemorrhage in September 2024, 3 years after diagnosis, from a cause considered unrelated to the lymphoma (**Figure 3**).

The patient was previously healthy with no chronic diseases such as hypertension, diabetes, or coronary heart disease. She had no history of infectious diseases such as hepatitis or tuberculosis, no history of surgery, trauma, or blood transfusion, and no history of food or drug allergies. There was no long-term use of special medications before onset. She had no history of smoking or alcohol consumption, and no family history of malignancy or autoimmune diseases.

### Ethics statement

Written informed consent was obtained from the patient. This study was conducted in accordance with the Declaration of Helsinki (1975). The study was approved by the Ethics Committee of Hebei General Hospital (No. 2025-339).

## Discussion

Primary diffuse large B-cell lymphoma (DLBCL) of the skeletal muscle is an extremely rare subtype of extranodal non-Hodgkin lymphoma (NHL), accounting for less than 0.1% of all extranodal lymphomas (8–10). According to the literature, the most common site is the skeletal muscle of the lower extremities, but involvement can also occur in special muscle groups such as the masticatory muscle and psoas major muscle. Most reported cases occur in elderly patients, with a median age of 70 years (9, 10). To the best of our knowledge, primary DLBCL of the rectus abdominis muscle has not been previously reported in the literature. Here, we report an 85-year-old female patient with a lesion located in the right rectus abdominis muscle. The diagnosis and treatment of this case highlight the complexity of imaging diagnosis for this disease and the decisive role of individualized treatment and pathological biopsy in optimizing the therapeutic strategy.

Although DLBCL is extranodal in approximately 25–40% of cases, primary nesting within skeletal muscle is exceptionally rare. The skeletal-muscle microenvironment is biologically inhospitable to malignant lymphocyte expansion: continuous mechanical strain disrupts stable intercellular contacts, the resident lymphocyte population is sparse, native lymphatic structures are scarce, and the cytokine milieu dominated by myokines such as IL-6, IL-15 and irisin is generally pro-inflammatory but not specifically pro-lymphomagenic. These factors are thought to underlie the very low incidence of primary skeletal-muscle DLBCL and explain why, when it does occur, it is often associated with focal architectural disruption (e.g., infiltrative growth along fascicular planes rather than expansile mass formation), as observed in our case.

Clinically, primary lymphoma of the skeletal muscle lacks specific manifestations. It usually presents as an insidious, ill-defined mass, which may be accompanied by local pain or tenderness, and some patients present with fever or nerve compression symptoms. Computed tomography (CT) or magnetic resonance imaging (MRI) mostly shows ill-defined soft-tissue masses. Contrast-enhanced ultrasound may reveal peripheral heterogeneous enhancement with central necrosis, but these findings are nonspecific (6, 7). Although the present patient presented with a right lower abdominal wall mass and aggravated pain, the advanced age of 85 years and unremarkable medical history easily led to a potential misdiagnosis as more common soft-tissue lesions, such as benign tumours, muscular abscesses, abdominal wall soft-tissue sarcomas, or hematomas. In this case, imaging revealed a mass in the abdominal wall muscular layer with an unclear boundary from the small intestine. However, pathological examination following needle biopsy finally confirmed the lesion as primary lymphoma of the abdominal wall muscle. Such imaging features may be related to the infiltrative growth pattern of lymphoma cells along tissue spaces rather than expansive growth.

In our patient, the principal differential diagnoses considered were soft-tissue sarcoma, desmoid tumour, intramuscular abscess, organising haematoma, inflammatory myofibroblastic tumour, and metastatic carcinoma to the abdominal wall. Soft-tissue sarcoma was considered less likely because the lesion was infiltrative rather than expansile, lacked the typical heterogeneous necrotic core seen in high-grade sarcomas, and showed only moderate (rather than markedly intense) enhancement; tumour markers and clinical history did not suggest metastatic disease. A desmoid tumour was considered unlikely given the patient’s age, the absence of prior abdominal surgery or pregnancy, and the rapid symptomatic progression. Intramuscular abscess was excluded by the absence of fever, normal white-cell count and CRP, lack of fluid component on contrast-enhanced CT, and absence of overlying skin changes. Organising haematoma was excluded by the absence of trauma or anticoagulant exposure, the progressive (rather than involutional) course over two weeks, and the solid (rather than fluid-density) appearance on CT. Inflammatory myofibroblastic tumour and metastatic carcinoma were ultimately excluded by histopathology and immunohistochemistry (CKpan negative, ALK negative, EMA negative, CD20 strongly positive). The infiltrative growth pattern observed on imaging may reflect the propensity of lymphoma cells to spread along tissue planes rather than to form expansile masses.

Due to the extreme rarity of such cases, misdiagnosis and inappropriate treatment are common. For instance, some cases were initially misdiagnosed as muscular abscesses and underwent incision and drainage, but the condition persisted without improvement, and the final diagnosis of DLBCL was established only by pathological examination (11). In addition, several cases of primary DLBCL of the gingiva with muscular invasion have been reported in the literature (12). These findings suggest that lymphoma should be considered in the differential diagnosis when dealing with deep abdominal-wall soft-tissue masses that exhibit atypical imaging features (e.g., infiltrative growth, hypervascularity disproportionate to size, lack of necrosis on enhanced CT) or do not align with common pathologies such as desmoid tumour, sarcoma, abscess, or haematoma. In this case, because CT showed a close relationship between the lesion and the intestine, surgeons timely postponed surgical resection and performed ultrasound-guided needle biopsy instead. This decision avoided inappropriate treatment and reduced unnecessary surgical trauma, which was especially critical for an 85-year-old patient with potentially impaired physiological reserve.

More broadly, contemporary geriatric oncology principles strongly support an “image then biopsy then resect” pathway for deep abdominal-wall masses in patients aged 80 years or older. Empirical surgical exploration in this population carries substantial risk of postoperative pulmonary complications, prolonged ileus, wound dehiscence, and cognitive decline, while offering little diagnostic advantage over modern image-guided core-needle biopsy. The favourable course of our patient — who avoided a potentially morbid laparotomy after the multidisciplinary team paused, obtained tissue diagnosis, and confirmed a chemosensitive lymphoma — supports the broader applicability of this conservative diagnostic algorithm in elderly patients with infiltrative abdominal-wall lesions.

Regarding imaging diagnosis, ultrasound, CT, and MRI have respective advantages in detecting skeletal muscle lymphoma. Conventional ultrasound and CT can identify solid intramuscular masses, but qualitative diagnosis is often difficult. Contrast-enhanced ultrasound (CEUS) shows relatively characteristic features in primary DLBCL of the psoas major muscle, typically presenting as heterogeneous hyperenhancement at the periphery of the lesion with non-enhanced necrotic areas in the centre. In comparison, MRI has superior soft-tissue resolution and can more clearly delineate the tumour extent and its relationship with surrounding tissues (13). In retrospect, a dedicated abdominal MRI would have provided better delineation of the lesion from adjacent bowel loops and is the recommended next-line imaging modality in similar future cases; in our patient, MRI was not pursued because the lesion had already been clearly localised by contrast-enhanced CT and CEUS, biopsy was already planned, and the patient declined further imaging on financial grounds. Furthermore, positron emission tomography/CT (PET/CT), as a functional imaging modality, exhibits unique advantages in the diagnosis, staging, and response assessment of DLBCL by accurately detecting high metabolic activity of the lesion (14). Nevertheless, given that some extranodal DLBCLs lack specificity and display distinct immunophenotypes, definite diagnosis usually cannot be achieved by imaging alone and must be combined with pathological and immunohistochemical analyses (15). In our patient, PET/CT and bone-marrow biopsy were declined; comprehensive conventional staging (contrast-enhanced chest/abdominal/pelvic CT, brain MRI, normal CBC/LDH/β2-microglobulin and peripheral-blood flow cytometry) supports — but does not definitively prove — the working diagnosis of localised primary extranodal disease.

As a systemic disease, the first-line treatment for DLBCL is comprehensive therapy dominated by chemotherapy rather than simple local surgical resection. Hasty extensive resection not only fails to achieve radical cure but may also delay the optimal time window for systemic therapy due to poor wound healing. Pathological and immunohistochemical analyses represent the gold standard for diagnosis and prognostic evaluation. The immunophenotype of this case was consistent with the non-germinal centre B-cell (non-GCB) subtype (MUM1+, CD10–), with a high Ki-67 proliferation index of approximately 90%, combined with expression of c-MYC (approximately 40%+) and Bcl-2 (+), indicating highly aggressive behaviour and a relatively poor prognosis. High expression of P53 (approximately 70%+) further suggests potential genetic complexity of tumour cells, which may increase the risk of chemoresistance.

However, DLBCL is a curable disease, especially when diagnosed at an early stage. The current standard regimen includes rituximab (anti-CD20 monoclonal antibody) combined with chemotherapy, such as cyclophosphamide, doxorubicin, vincristine, and prednisone (the R-CHOP regimen), which has significantly improved remission rates (16, 17). In very elderly or cardiovascularly fragile patients, R-THP-COP in which pirarubicin (THP), an anthracycline analogue with reduced cardiotoxicity, replaces doxorubicin has been shown in both phase II prospective and retrospective comparative studies to achieve response and survival outcomes comparable to R-CHOP, with a more favourable cardiac safety profile (18–20); this rationale guided regimen selection in our 85-year-old patient. Nevertheless, high-level evidence for primary skeletal muscle DLBCL is still lacking. In this case, the patient achieved long-term remission after 4 cycles of R-THP-COP therapy, providing a reference for the treatment of similar cases in the future. However, due to the absence of large-sample clinical trials, the level of evidence is relatively low and only for clinical reference.

At the diagnostic level, the combination of MRI and 18F-FDG PET/CT is valuable for evaluating lesion extent and metabolic activity, whereas histopathological examination combined with immunohistochemical analysis remains the gold standard for definite diagnosis. Particularly, identification of the non-GCB subtype and special molecular phenotypes is crucial for designing precise therapeutic strategies. Regarding treatment, the R-CHOP-based regimen is currently the mainstay to improve patient prognosis, with R-THP-COP serving as a reasonable alternative for elderly or cardiovascularly fragile patients. Meanwhile, individualized adjustment according to the patient’s physical condition is important for optimizing therapeutic strategies and supportive care. Future studies should focus on accumulating larger clinical cohorts, exploring specific molecular genetic alterations, and developing targeted agents against specific molecular targets, so as to further improve the cure rate and long-term quality of life of patients with this rare disease.

In summary, primary DLBCL of the skeletal muscle, as an extremely rare extranodal lymphoma, demonstrates highly complex and heterogeneous clinical, pathological, and biological characteristics. By integrating the available literature and clinical data, this study systematically elaborates the definition, epidemiology, unique pathogenesis, and clinical challenges in the diagnosis and treatment of primary muscular DLBCL. The disease mostly affects middle-aged and elderly individuals and often follows an aggressive clinical course. Due to the lack of specific clinical symptoms and imaging findings, it is easily misdiagnosed as soft-tissue sarcoma or muscular abscess, leading to delayed diagnosis and inappropriate treatment. Therefore, clinicians should consider including lymphoma in the differential diagnosis when dealing with deep abdominal-wall soft-tissue masses that exhibit atypical imaging features or do not align with common pathologies, and should favour an image-guided biopsy-first diagnostic pathway in elderly patients before committing to surgical resection.

### Limitations

Several limitations of this report should be acknowledged. First, baseline and post-treatment 18F-FDG PET/CT currently the standard for staging and response assessment in DLBCL were not performed because the patient declined them on financial grounds; without PET/CT, occult low-volume nodal or bone-marrow involvement cannot be excluded with certainty, and our designation of “primary” rectus-abdominis DLBCL should therefore be regarded as a clinicoradiological inference rather than a definitive PET-confirmed diagnosis. Second, bone marrow biopsy also part of standard staging was not obtained for the same reason. Third, a dedicated abdominal MRI was not performed; in retrospect MRI would have provided better soft-tissue characterisation and is recommended in similar future cases. Fourth, post-treatment “complete response” was assessed by contrast-enhanced CT only and represents an anatomical rather than metabolic response. Fifth, after CR, structured imaging surveillance was not performed; the patient declined further hospital visits and was followed by intermittent telephone contact every 3–6 months until her death from cerebral haemorrhage three years after diagnosis. Telephone follow-up can confirm only the absence of reported clinical symptoms, not the absence of asymptomatic disease recurrence. Finally, conclusions drawn from a single case cannot validate any specific therapeutic regimen for this disease entity. The favourable outcome retrospectively supports the clinical decision taken for this specific patient (image-guided biopsy followed by an attenuated, less cardiotoxic anthracycline-based regimen) but does not, by itself, validate this approach more broadly; confirmation in larger series is required.

## Data Availability

The original contributions presented in the study are included in the article/**Supplementary Material**. Further inquiries can be directed to the corresponding authors.
