# Supplementary figures and images for: Primary diffuse large B-cell lymphoma of the rectus abdominis muscle: a presumed primary case report and literature review
Source: Front Oncol. 2026 Jun 2;16:1837952. doi: 10.3389/fonc.2026.1837952 (PMC13268874; doi:10.3389/fonc.2026.1837952)

Immunohistochemistry results, 200x magnification

S1-HE
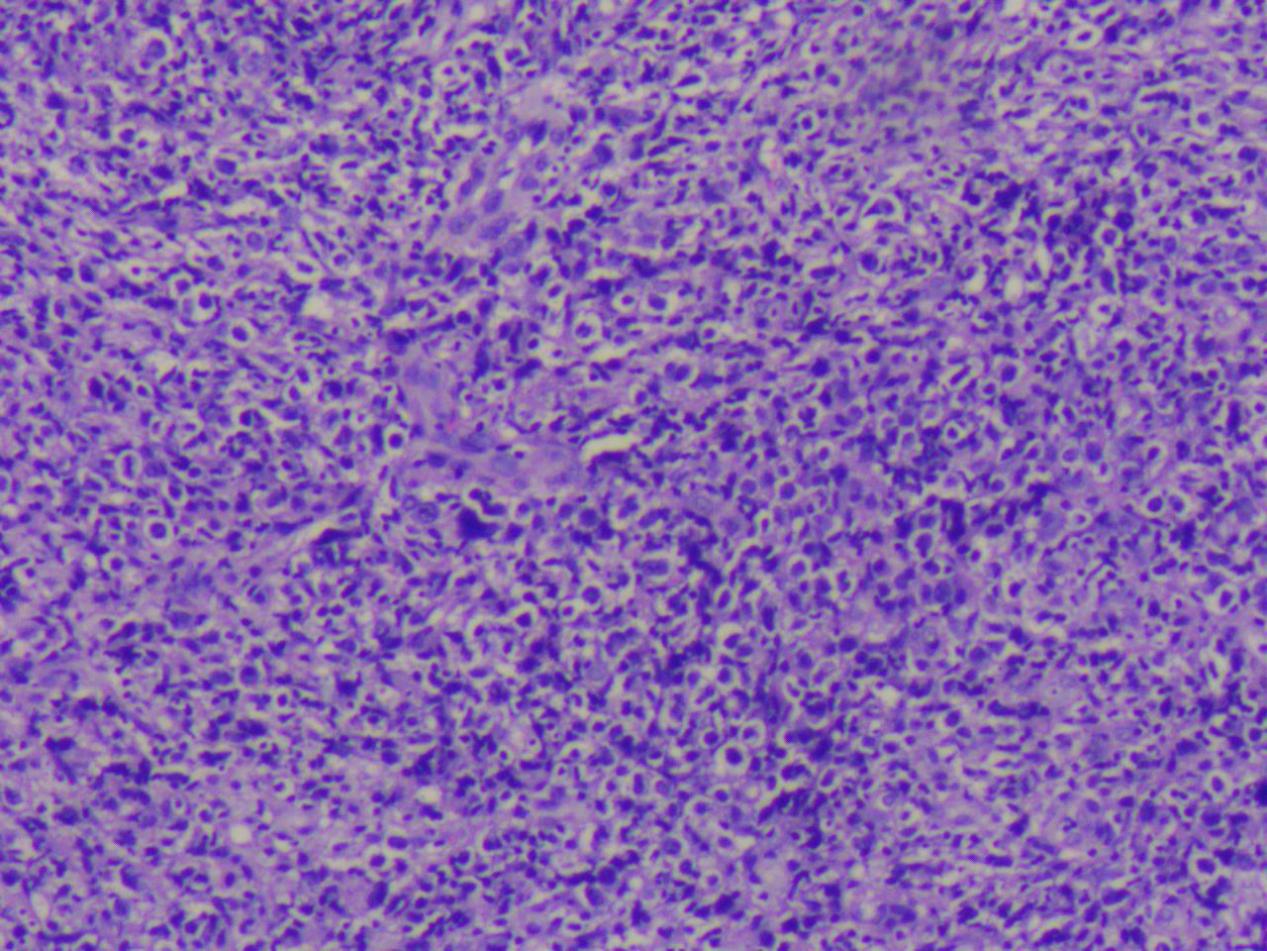


S2-CD20


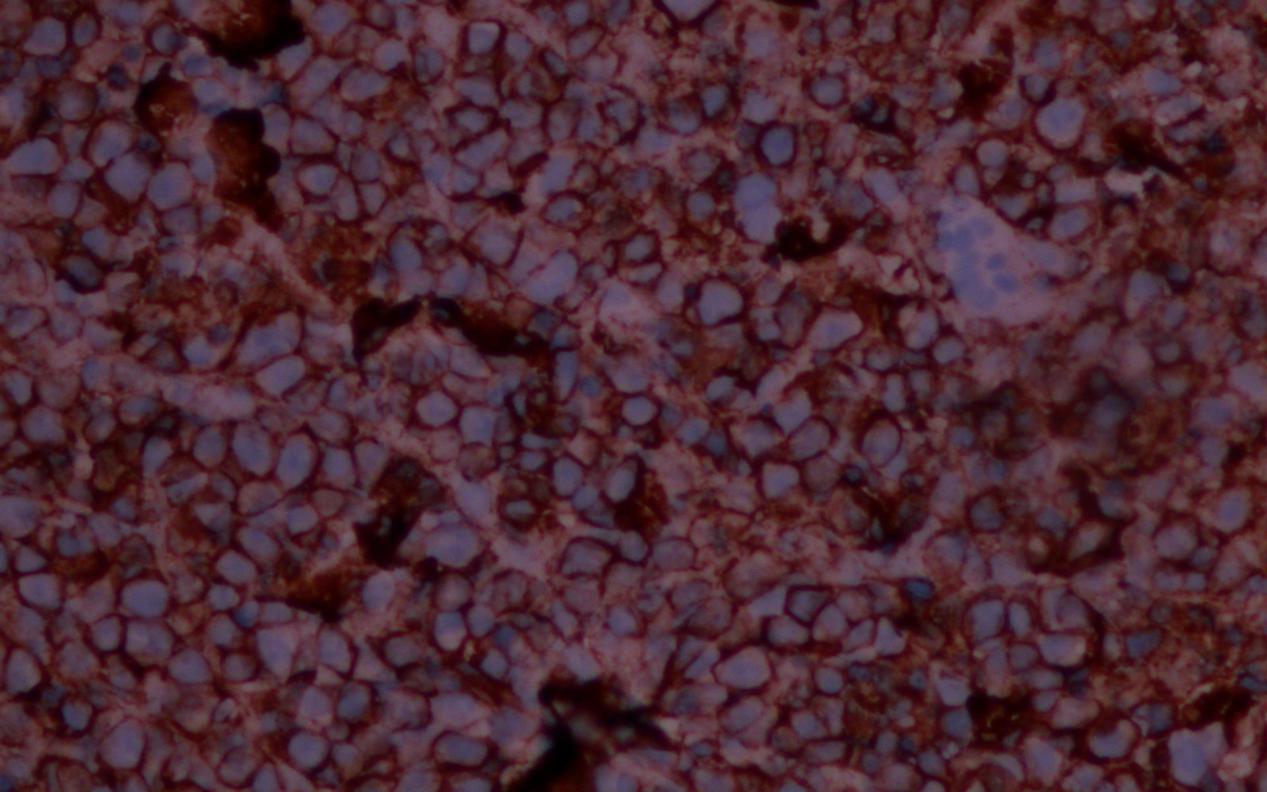


S3-CD3


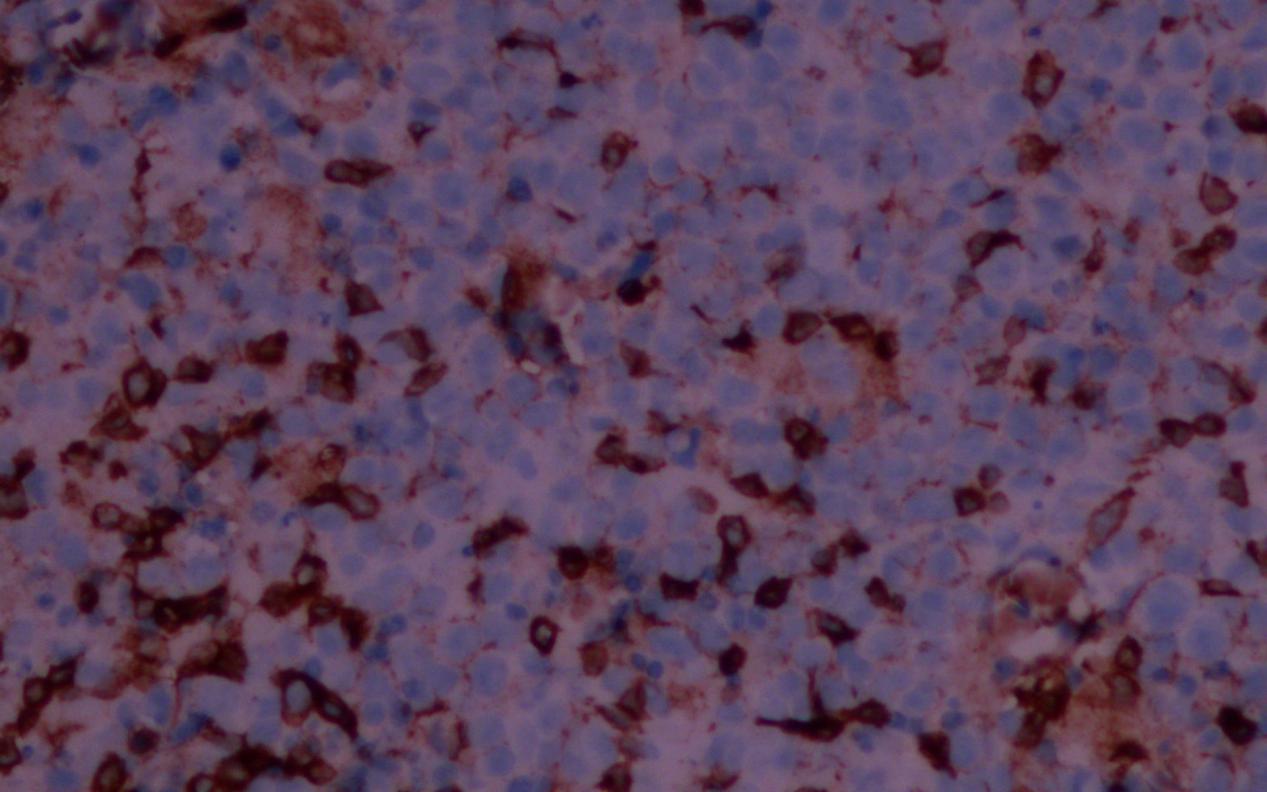


S4-CD21


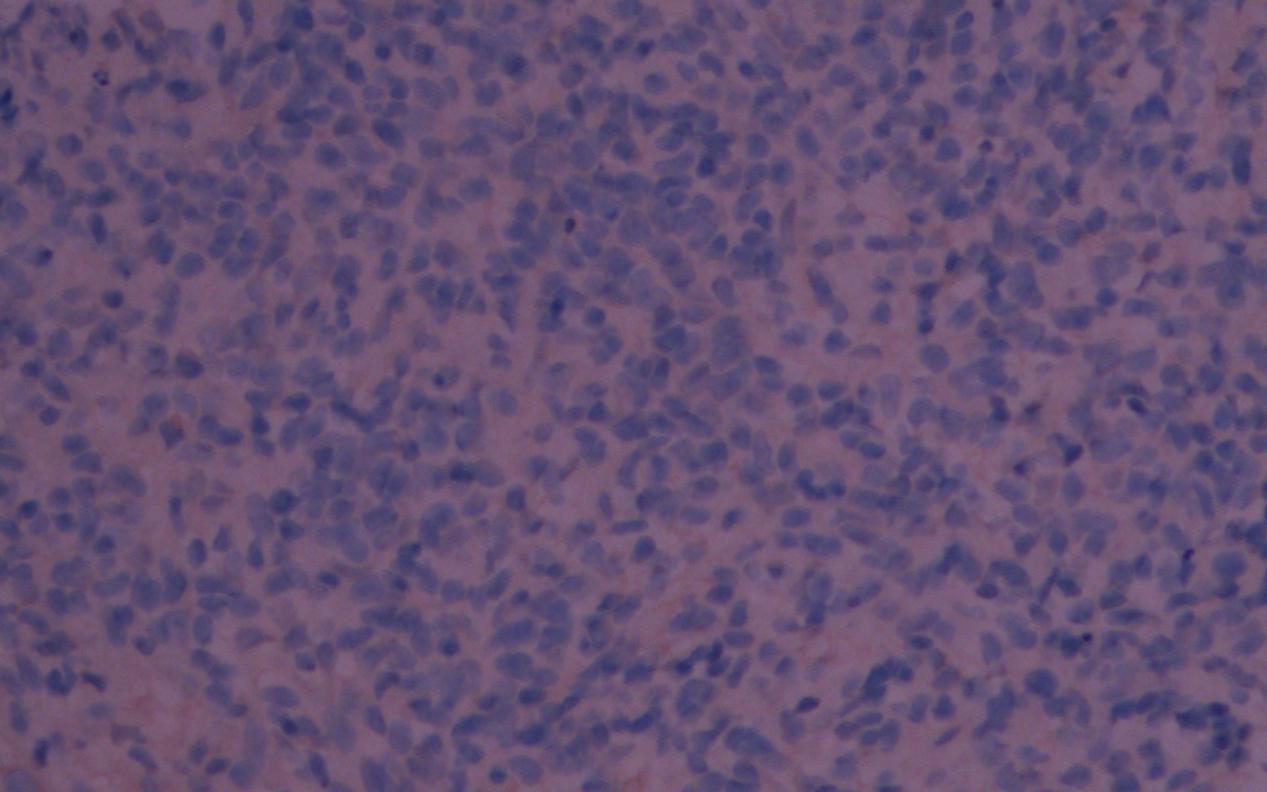


S5-ki67


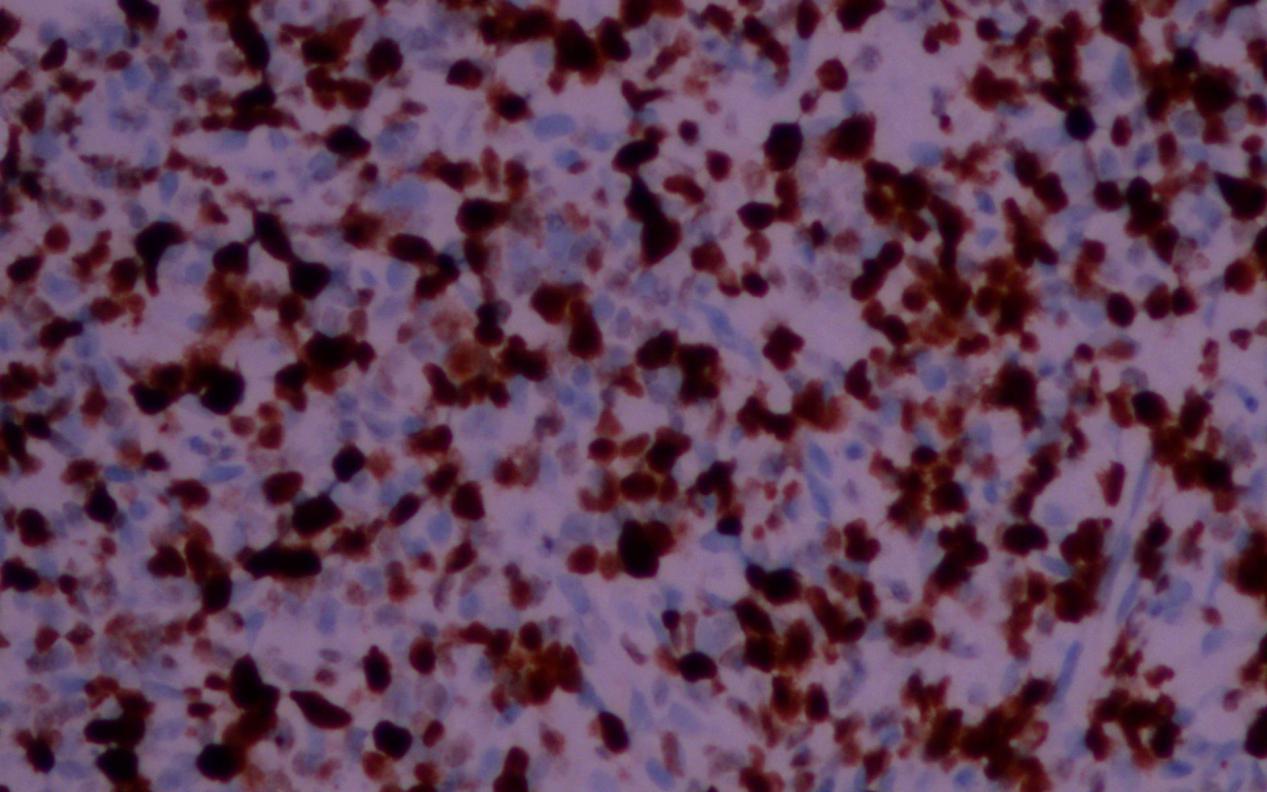


S6-MUM1


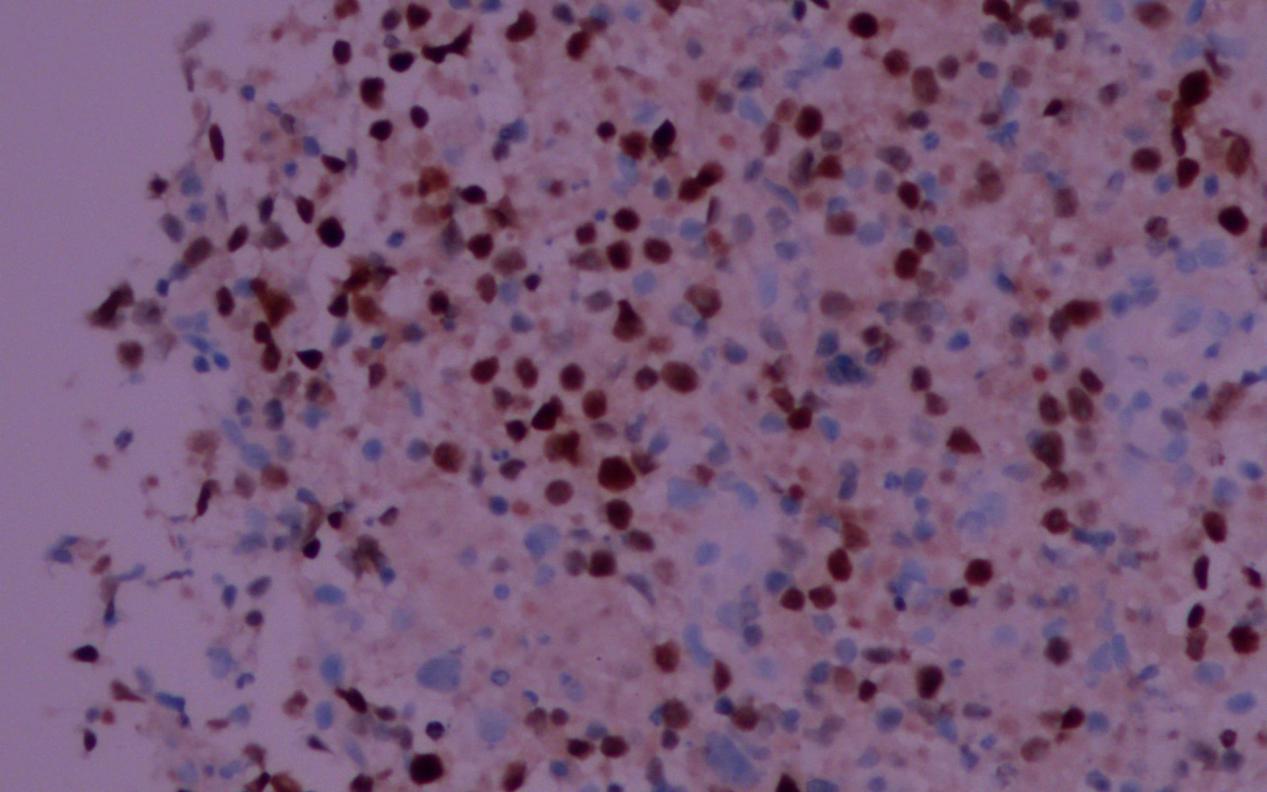


S7-blc6


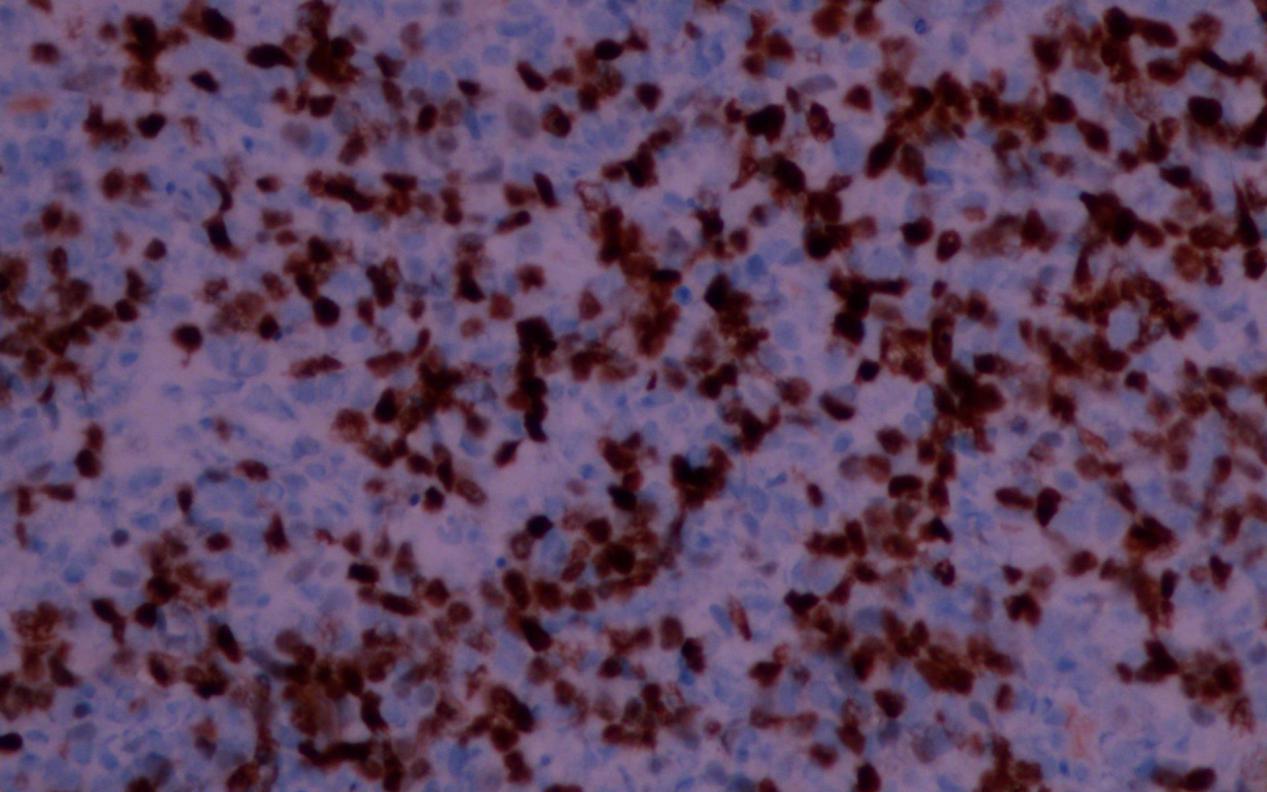


S8-bcl2
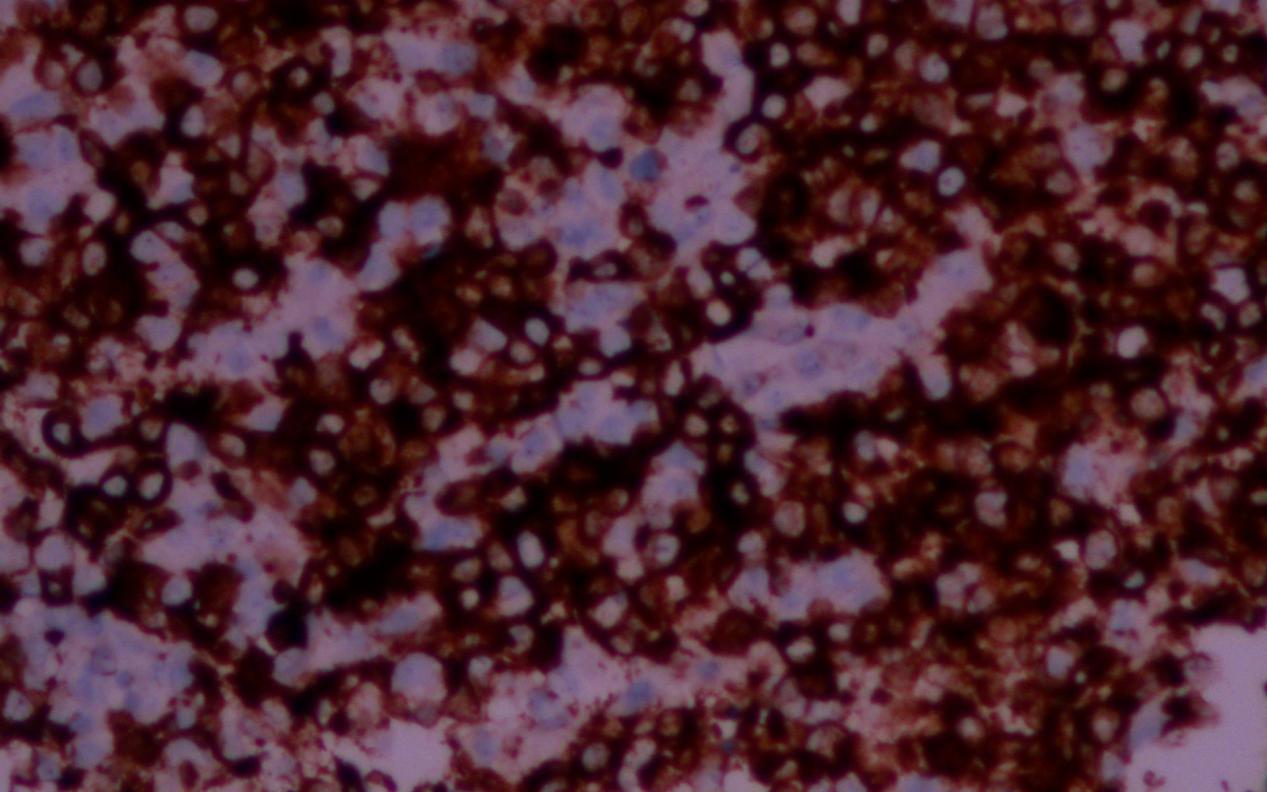


S9-CD10
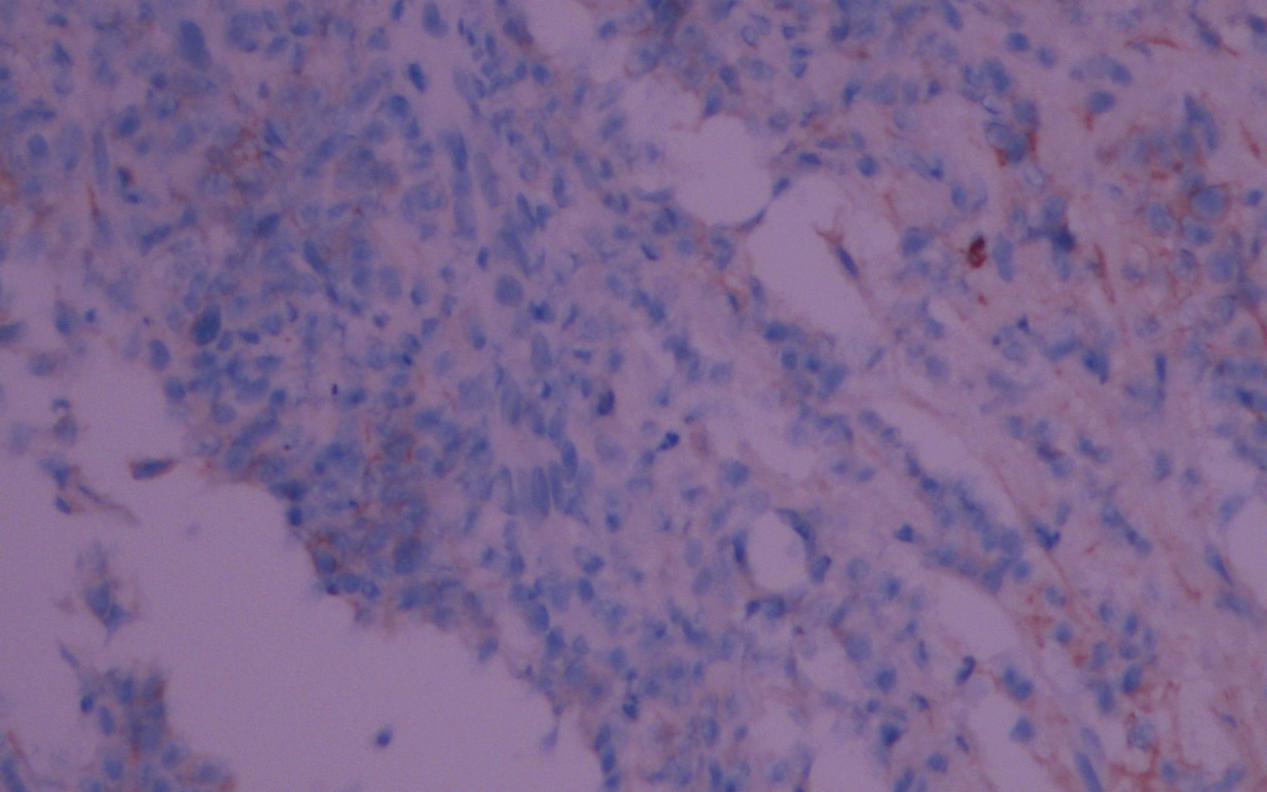

Supplement: Supplementary file 1 [file DataSheet1.docx]
